# Predicting hepatic encephalopathy in patients with cirrhosis: A UK population–based study and validation of risk scores

**DOI:** 10.1097/HC9.0000000000000307

**Published:** 2023-11-06

**Authors:** Bethan I. Jones, Cerys A. Jenkins, Daniel Murphy, James Orr, Andrew Yeoman, Ellen R. Hubbuck, Ben R. Heywood, Craig J. Currie

**Affiliations:** 1 Global Pharmacoepidemiology, Human Data Sciences, Cardiff, UK; 2 Market Access and Government Affairs, Norgine Pharmaceuticals Limited, Uxbridge, UK; 3 Gwent Liver Unit, Hepatology Department, University Hospitals Bristol and Weston, Bristol, UK; 4 Gwent Liver Unit, Aneurin Bevan University Health Board, Royal Gwent Hospital, Newport, Wales, UK; 5 Institute of Population Health, School of Medicine, Cardiff University, Cardiff, UK

## Abstract

**Background:**

HE is a common neurologic complication in cirrhosis associated with substantial disease and economic burden. HE symptoms are nonspecific and there are limited ways of identifying patients with cirrhosis at high risk of later developing HE. A risk score was previously developed to identify patients at risk of developing HE in a predominately male US cohort. Here, we evaluated the performance of the HE risk scores in a UK cohort study.

**Methods:**

Health care records from Clinical Practice Research Datalink and linked Hospital Episode Statistics were used to select patients with cirrhosis who were diagnosed with HE, confirmed by a diagnosis code for HE or a rifaximin-α prescription. The index date was the date of incident cirrhosis. The study period was from January 2003 to June 2019.

**Results:**

A total of 40,809 patients with cirrhosis were selected in the UK cohort, of whom 59% were male. A total of 1561 patients were diagnosed with HE. Applying the UK cohort to the baseline sensitivity risk cutoff (≥−11) from the US cohort provided a sensitivity of 92% and a negative predictive value of 99%. Within a longitudinal model, applying a sensitivity cutoff of ≥−3 to this cohort gave a sensitivity of 89% and a negative predictive value of 99%.

**Conclusions:**

Using data from the UK, the previously developed HE risk scores were found to be reliable for selecting those most likely to progress to HE in patients with liver cirrhosis. Despite the HE risk scores originally being estimated using the data from a predominately male US cohort, the scores were validated and found to be generalizable to female patients.

## INTRODUCTION

Cirrhosis inhibits the normal functioning of the liver, which can lead to complications of portal hypertension, including HE.^1,2^ HE encompasses a wide range of neurological symptoms from subtle cognitive impairment to coma.^3^ HE with minimal symptoms (covert HE) occurs in 20%–80% of patients with cirrhosis.^4–6^ Fully symptomatic overt HE occurs in 30%–40% of those with cirrhosis at some point during their clinical course.^7,8^

HE is associated with increased patient mortality risk, more frequent hospitalization episodes, lower quality of life, severe disease, and a large economic burden from decreased productivity.^9–12^ There are limited data to describe the characteristics of patients with cirrhosis at the highest risk of developing HE, or how their risk changes following an improvement in liver function. Furthermore, limited data exist describing the effects of various medications on HE risk. The ability to risk-stratify patients would allow for better-informed resource utilization, help better manage the local burden of HE, and closely monitor those at the highest risk.

Using data from the Veterans Administration from Michigan, Indiana, and Ohio,^13^ Tapper and colleagues studied the demographic, clinical, laboratory, and pharmacy characteristics of this population to identify factors associated with progression to HE. They identified a cohort of 1979 patients with cirrhosis without HE. The study reported risk factors for HE development including higher bilirubin and nonselective beta-blocker use, while higher albumin and statin use were found to be protective.^13^ Model effects were validated and converted into a risk score by summing risk points accumulated by each risk factor (beta-blocker use, statin use, total bilirubin, and albumin). Tertiles of risk scores were calculated from all patients’ risk scores and were then used to classify patients into low-risk, medium-risk, and high-risk groups.

Two risk models were constructed, a baseline model giving a 5-year risk of developing HE and a longitudinal model giving a 1-year risk based on the patient’s most recent clinical data. The risk scores were successful in predicting patients with cirrhosis at risk of developing HE. Minimum sensitivity and specificity cutoffs of 90% were applied to the risk models to give risk score cutoffs to maximize sensitivity and specificity. In the baseline model, a risk score cutoff of ≥−11 gave a 90.7% sensitivity and a cutoff of ≥27 provided a 91.2% specificity. In the longitudinal model, a cutoff of ≥−3 gave a sensitivity of 90.3%, while a cutoff of ≥19 gave a specificity of 90.6%.

The risk models enabled the risk stratification of patients with cirrhosis into low-risk, medium-risk, and high-risk groups (calculated using tertiles of risk) based on routinely available data. The risk model cutoff values to stratify patients were changed within the study to maximize sensitivity or specificity depending on the clinical setting and produced high values. However, the study was conducted in a specific population of predominantly male patients from the United States, and therefore may not be generalizable. This study was an external validation of the developed HE risk score using UK data consisting of both male and female patients.

## METHODS

### Data source

The data for this study were retrieved from the Clinical Practice Research Datalink (CPRD) and Hospital Episode Statistics (HES) databases. CPRD is a longitudinal, anonymized research database derived from nearly 700 primary care practices in the United Kingdom. CPRD consists of 2 separate databases: CPRD GOLD and CPRD Aurum. CPRD can be linked to other data sources, including HES data sets, which provide data on all inpatient and outpatient contacts occurring within National Health Service hospitals in England. Approximately 54% of GOLD practices and roughly 93% of Aurum practices available in the January 2019 build are eligible for linkage to HES.^14^ Diagnostic information is recorded using the Read code classification and Systematized Nomenclature of Medicine codes in CPRD GOLD and Aurum, respectively. HES inpatient data are recorded using the International Classification of Diseases 10 (ICD-10) classification. CPRD GOLD and Aurum were used in combination for this analysis, with any patients who transferred from GOLD to Aurum being removed from the GOLD dataset to avoid duplication. CPRD data quality is ascribed in CPRD by flags applied at a practice and patient level. Practices are deemed to be “up to standard” when the data recorded for each practice are within the range expected; however, this is only currently available for GOLD data. Patients in GOLD and Aurum are classified as acceptable research quality if they have a valid coding for gender, have a valid birth year with no prior activity recorded before this date, and have an age of <115 at the last data collection point. They must also be permanently registered at the practice. This study only used patients who were deemed as acceptable research quality.

Data from CPRD are obtained under the license from the UK Medicines and Health care products Regulatory Agency. This study was conducted under CPRD Independent Scientific Advisory Committee approval (ISAC 19_179). This study is based in part on data from the Clinical Practice Research Datalink obtained under licence from the UK Medicines and Healthcare products Regulatory Agency. The data is provided by patients and collected by the NHS as part of their care and support.

HES data (Copyright © 2023), re-used with the permission of The Health & Social Care Information Centre. All rights reserved.

The interpretation and conclusions contained in this study are those of the author/s alone.

### Patient selection

Patients with cirrhosis for this study were selected from primary and secondary care data by primary care codes, ICD-10 codes, and Operating Procedure Codes Supplement Classification of Interventions and Procedures codes. Patients with HE were included in the analysis if they had a primary care diagnosis code for HE or a prescription in the therapy table for rifaximin-α. The index date was set to the earliest recorded diagnosis of cirrhosis in CPRD (incident cirrhosis event). The study period was from 1^st^ January 2003 to the earliest of death, end of CPRD follow-up, liver transplant date, or 5 years after diagnosis.

Patients were included in all analyses if they met the following inclusion criteria: 18 years of age or above, first diagnosis date ≥2003, and for GOLD patients index date had to be after CPRD up to standard date. Patients were excluded if they had received a liver transplant prior to the index date or a cirrhosis diagnosis date after HE diagnosis date.

The primary analysis was defining HE as a prescription of rifaximin-α 550 mg was used as a proxy for a HE diagnosis within this study. Therefore, a sensitivity analysis was conducted to examine the effect of the proxy inclusion on the performance of the risk stratification scores for predicting HE onset in the UK population. Sensitivity analysis was conducted using only patients with HE selected by primary care diagnosis codes. The index date for the sensitivity analysis was the earliest recorded diagnosis date with a HE CPRD read code or Systematized Nomenclature of Medicine code.

Patients with HE can be treated with lactulose alone; however, there are many other reasons why a lactulose script could be prescribed. Investigation into the use of lactulose as a marker of the diagnosis of HE was conducted via a further sensitivity analysis. The sensitivity analysis was conducted including lactulose prescription codes as a diagnosis for HE or a diagnosis of HE.

### Study outcomes

The primary objective of this study was to validate the HE risk scores by using them to predict the onset of HE within the UK population. The secondary objective was to generate the risk score when subjects were stratified by gender and by age group to ensure generalizability to the entire UK adult population. Finally, risk score cutoffs were varied to achieve a minimum sensitivity of 80% for the UK cohort. Patients were followed until the earliest of death, end of CPRD follow-up, liver transplant date, or 5 years after diagnosis. Descriptive statistics were compiled for the population with cirrhosis, the population with HE, and the population without HE.

### Statistical analysis

Data extraction was completed in SQL server, and base R (version 4.1.1) was used for all statistical analysis. Baseline characteristics were reported for the population with cirrhosis and both the patients with and without HE subcohorts. Categorical data were calculated as counts (n) and proportions (%). For continuous data, summary statistics were calculated. The number and proportions of missing data were reported as a separate category for each variable. Differences between groups were examined using appropriate statistical tests (Wilcoxon rank-sum test for continuous data and χ^2^ for categorical data). Baseline characteristic variables were selected to mirror those reported in the US cohort study conducted by Tapper et al.^13^

The baseline HE risk score gives a 5-year risk of a patient developing HE from the point of cirrhosis diagnosis. A person’s risk score was calculated by summing the number of points accumulated by each risk factor (beta-blocker use, statin use, total bilirubin, and albumin). The number of points assigned for each risk factor varies and is based on the risk score points in the baseline risk score.^13^ A patient was classed as high risk (≥21 points), intermediate risk (−9 to 20 points), or low risk (<−10).

The longitudinal HE risk score provides a 1-year risk of HE for any given patient based on their most recent clinical parameters. This method treats each person from index diagnosis (cirrhosis) as a new patient with updated clinical parameters in each person-year. A person’s 1-year risk score was calculated by summing the points from each risk factor variable based on those assigned in the longitudinal HE risk score.^13^ A patient is classed as high risk (≥21 points), intermediate risk (1–20), or low risk (≤0). The last observation carried forward was used to impute missing values where possible.

For both the baseline and longitudinal scores from the original US cohort, sensitivity and specificity cutoff values were selected to achieve minimum values of 90%. Confusion matrices were produced to calculate the sensitivity, specificity, negative predictive value (NPV), and positive predictive value (PPV) for each risk score cutoff selected for comparison to the HE risk scores (Supplemental Figure S1, http://links.lww.com/HC9/A628). The true positive rate was calculated by taking the proportion of patients where the risk score predicted HE, who also had a read code or prescription confirming HE. The performance of the longitudinal HE risk score was validated on the same person up to 5 times, with the date of data collection being on an annual basis from the index date. The risk scores for all the patients were calculated using their latest clinical parameters at the point of presentation. Their 1-year risk was calculated using the longitudinal HE risk score.

The performance of the risk scores was also evaluated when the patients were stratified by gender. The risk score cutoff points were varied from those used in the US cohort to achieve a minimum sensitivity of 80% within the UK cohort. As a sensitivity analysis, validation was repeated across both HE risk scores with only patients with HE selected by primary care diagnosis codes.

Both the baseline and longitudinal performances were evaluated using the AUC. The integral of the receiver operating characteristic curve was used to calculate the AUC. A higher AUC value indicates a better discriminatory performance of the model. The AUC values were compared against Tapper AUC values.

## RESULTS

**FIGURE 1:**
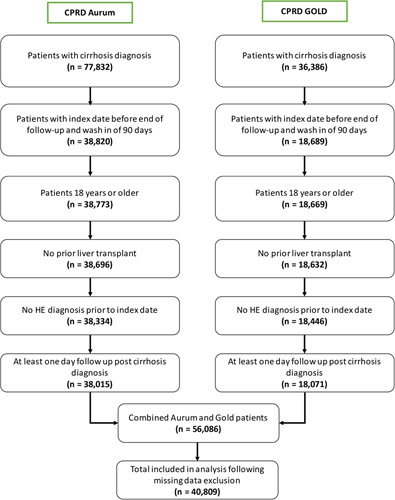
Attrition flow diagram combining CPRD Aurum and CPRD GOLD data sets. Abbreviation: CPRD, Clinical Practice Research Datalink.

A total of 56,086 UK patients were selected from the CPRD GOLD and Aurum, who met the criteria for inclusion in the study (Figure 1). Following the removal of duplicated patients and the patients with missing data for the variables required to calculate the risk score, the study population included in the analysis was reduced to 40,809 patients. Of these, 1,561 went on to be diagnosed with HE in the following 5 years and 39,248 were not diagnosed with HE, of which 17,277 died.

### Baseline characteristics

Baseline characteristics are summarized in Table 1. Of the UK cohort with cirrhosis, 59% were male. The mean age of those with cirrhosis was 61 years (±14 y) with the >60 age group containing the highest proportion of patients (53%). Excluding the missing category, patients with a BMI classed as “obese” and “overweight” contained the greatest proportion of patients with 14% and 11%, respectively. Of the 1561 patients who developed HE, the baseline characteristics were largely similar to the main cohort with cirrhosis with 65% being male with a mean age of 60 years. A total of 39,248 patients were not diagnosed with HE. The mean bilirubin levels were greater in the cohort with HE compared to the cohort without HE, 2.9 and 1.9 mg/dL, respectively. Statin and beta-blocker uses were comparable between patients with and without HE, with 28% of the overall population with cirrhosis taking beta-blockers and 30% taking statins. Baseline characteristics for the sensitivity cohort (patients with primary care diagnosis codes only) are summarized in Supplemental Table S1, http://links.lww.com/HC9/A628. Baseline characteristics for the second sensitivity cohort (inclusion of lactulose codes) are summarized in Supplemental Table S3, http://links.lww.com/HC9/A628.

**TABLE 1:** Baseline characteristics

	Patients with cirrhosis	Developed HE	No HE	*p*
Total, n	40,809	1561	39,248	—
Age, (y)	—	—	—	<0.001
Mean (SD)	61.4 (13.7)	59.8 (11.5)	61.5 (13.8)	—
Median (IQR)	62.0 (52.0–71.0)	60.0 (52.0–68.0)	62.0 (52–72)	—
Gender, n (%)	—	—	—	<0.001
Male	24,107 (59.0)	1021 (65.0)	23,086 (59.0)	—
Female	16,702 (41.0)	540 (35.0)	16,162 (41.0)	—
BMI, kg/m^2^	—	—	—	<0.001
Mean (SD)	29.0 (6.8)	30.0 (6.5)	29.0 (6.8)	—
Median (IQR)	28.2 (24.2–32.9)	29.3 (25.5–34.1)	28.2 (24.1–32.9)	—
Albumin, g/dL	—	—	—	<0.001
Mean (SD)	3.6 (0.7)	3.3 (0.6)	3.6 (0.7)	—
Median (IQR)	3.7 (3.1–4.1)	3.3 (2.8–3.7)	3.7 (3.1–4.1)	—
Bilirubin, mg/dL	—	—	—	<0.001
Mean (SD)	2.0 (3.3)	2.9 (3.7)	1.9 (3.2)	—
Median (IQR)	0.9 (0.6–1.9)	1.8 (1.0–3.3)	0.9 (0.5–1.8)	—
Beta-blockers, n (%)	—	—	—	0.016
Yes	11,277 (27.6)	473 (30.3)	10,804 (27.5)	—
No	29,532 (72.4)	1088 (69.7)	28,444 (72.5)	—
Statins, n (%)	—	—	—	0.4
Yes	12,421 (30.4)	489 (31.3)	11,932 (30.4)	—
No	28,388 (69.6)	1072 (68.7)	27,316 (69.6)	—
Age categories, n (%)	—	—	—	<0.001
Age 18–30	477 (1.2)	7 (0.5)	470 (1.2)	—
Age 31–40	2212 (5.4)	74 (4.7)	2138 (5.5)	—
Age 41–50	6432 (15.8)	258 (16.5)	6174 (15.7)	—
Age 51–60	10,062 (24.7)	442 (28.3)	9620 (24.5)	—
Age >60	21,626 (53.0)	780 (50.0)	20,846 (53.1)	—
BMI categories, n (%)	—	—	—	<0.001
Underweight	413 (1.0)	11 (0.7)	402 (1.0)	—
Normal weight	3905 (9.6)	130 (8.3)	3775 (9.6)	—
Overweight	4558 (11.2)	187 (12.0)	4371 (11.1)	—
Obese	5882 (14.4)	297 (19.0)	5585 (14.2)	—
Missing	26,051 (63.8)	936 (60.0)	25,115 (64.0)	
Follow-up, (d)				
Mean (SD)	835.6 (685.0)	942.8 (589.2)	831.3 (688.2)	<0.001
Median (IQR)	671.0 (180.0–1581.0)	869.0 (435.0–1496.0)	661.0 (170.0–1588.0)	—

Abbreviations: BMI, body mass index; IQR, interquartile range.

### Baseline risk score validation

Patients were sorted via the HE risk scores into high-risk, medium-risk, and low-risk groups. The highest proportion of patients with cirrhosis had a risk score in the medium range of −9 to 20 (53%), with the lowest number of patients with cirrhosis receiving a risk score of ≥21 (19%) (Table 2). Of these patients grouped by risk score, the group with the highest proportion of patients with HE was ≥21 with 7% of patients developing HE. The group with the lowest number of patients who developed HE was the low-risk category, ≤−10 (2%).

**TABLE 2:** Proportion of UK patients in each risk score group

Risk groups	Cirrhosis (%)	Developed HE (%)
Baseline model (per patient)		
Low = ≤−10	28.4	1.7
Medium = −9 to 20	53.0	4.0
High = ≥21	18.6	6.6
Longitudinal model (per patient-year)		
Low = ≤0	51.9	1.0
Medium = 1–20	36.3	4.0
High = ≥21	11.8	8.3

**FIGURE 2:**
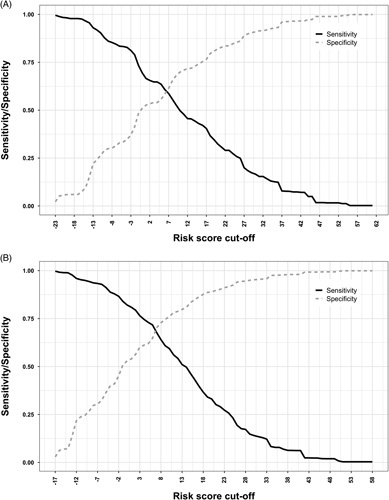
(A) Baseline model change in sensitivity and specificity as the cutoff is increased. (B) Longitudinal model change in sensitivity and specificity as cutoff is increased.

The AUC scores for the baseline model in the United Kingdom and United States were 0.58 and 0.68, respectively. The longitudinal model’s AUC scores were 0.64 for the United Kingdom and 0.73 for the United States. When using the cutoff of ≥−11 points, which was used in the US cohort to calculate a sensitivity of 91%, the sensitivity from our data is 92% with a specificity of 24% and a NPV of 99% (Table 3). When using the cutoff to achieve a specificity of 91% (≥27) from the US cohort, the specificity of the model for UK data was 86% with a sensitivity of 25%, an NPV of 97%, and a PPV of 7%. Varying the risk score value to reach a target sensitivity of >80% resulted in a cutoff at ≥−2, which produced a sensitivity of 81%, a specificity of 37%, an NPV of 98%, and a PPV of 4.9% (Figure 2A). The sensitivity cohort analysis using only patients with HE selected by primary care diagnosis codes produced comparable results to the primary analysis cohort (Supplemental Table S2, http://links.lww.com/HC9/A628). Further analysis into patients identified with HE with lactulose included produced AUC scores of 0.58 for the baseline model and 0.64 for the longitudinal model (Supplemental Table S4, http://links.lww.com/HC9/A628).

**TABLE 3:** Comparison of risk model performance for the baseline and longitudinal models

	Baseline model		Longitudinal model	
	US model	UK model	US model	UK model
AUC	0.68	0.58	0.73	0.64
Sensitivity cutoff (%)	≥−11	≥−11	≥−3	≥−3
Patients	—	76.5	—	60.9
Sensitivity	90.7	91.9	90.3	88.8
Specificity	—	24.1	—	39.9
Negative predictive value	—	98.7	—	99.2
Positive predictive value	—	4.6	—	4.3
Specificity cutoff (%)	≥27	≥27	≥19	≥19
Patients	—	14.3	—	13.0
Sensitivity	—	24.9	—	36.7
Specificity	91.2	86.1	90.6	87.7
Negative predictive value	—	96.6	—	97.9
Positive predictive value	—	6.6	—	8.3
>80% sensitivity cutoff (%)	—	≥−2	—	≥2
Patients	—	63.7	—	47
Sensitivity	—	81.2	—	80.9
Specificity	—	37.0	—	54.0
Negative predictive value	—	98.0	—	98.9
Positive predictive value	—	4.9	—	5.1

When stratifying patients by gender and using the cutoff of ≥−11, which achieved a minimum sensitivity of 90% in the predominately male US cohort, the UK female cohort gave the highest sensitivity reported (92%) and the male cohort also achieved a high sensitivity >91%. Using the cutoff of ≥27 used to achieve a minimum 90% specificity in the predominately male US cohort, the female cohort achieved a higher specificity than the male cohort (87%), with both cohorts achieving a high specificity overall >96% (Table 4).

**TABLE 4:** Risk model performance for the baseline model by gender

	Overall	Male	Female
Risk score cutoff: ≥−11 (%)			
Sensitivity	91.9	91.6	92.4
Specificity	24.1	23.9	24.4
Negative predictive value	98.7	98.5	99.0
Positive predictive value	4.6	5.1	3.9
Risk score cutoff: ≥27 (%)			
Sensitivity	24.9	24.7	25.2
Specificity	86.1	85.8	86.5
Negative predictive value	96.6	96.3	97.2
Positive predictive value	6.6	7.2	5.9

### Longitudinal risk score validation

The highest proportion of patients had a risk score of ≤0 (52%), with the lowest number of patients receiving a risk score of ≥21 (12%) (Table 2). Of these patients grouped by risk score, the group with the highest proportion of patients with HE was the patients with a risk score of ≥21 with 8% of the patients developing HE. The group with the lowest number of patients with HE was the group with a score of ≤0 (1%). When using the same cutoff used in the US cohort of ≥−3 points, at which they calculated a sensitivity of 90%, the sensitivity of the model was shown to be 89% in the UK cohort with a specificity of 40% (Table 3). The PPV was 4% and the NPV was 99%. When using the same cutoff used in the US cohort of ≥19 points to calculate a specificity of 91%, the specificity from the UK cohort was 88% with a sensitivity of 37%. The calculated PPV was 8% and the NPV was 98%.

Using a target sensitivity cutoff of >80% resulted in a risk score cutoff at ≥2, which produced a sensitivity of 81%, a specificity of 54%, a PPV of 5%, and an NPV of 99% (Figure 2B). The sensitivity cohort analysis using only patients with HE selected by primary care diagnosis codes produced comparable results to the primary analysis cohort (Supplemental Table S3, http://links.lww.com/HC9/A628).

When stratifying patients by gender and using a cutoff of ≥−3 (predominately male US cohort, 90% sensitivity), the male cohort had the highest sensitivity of 89% and the female cohort also achieved a good sensitivity of 86%. Using the cutoff of ≥19, the predominately male US cohort achieved 91% specificity; but when stratified by gender, the female cohort achieved a higher specificity of the 2 cohorts with a specificity of 90%, with the male cohort achieving 88% specificity (Table 5).

**TABLE 5:** Risk model performance for the longitudinal model by gender

	Overall	Male	Female
Risk score cutoff ≥−3 (%)			
Sensitivity	88.8	88.7	86.4
Specificity	39.9	40.6	41.5
Negative predictive value	99.2	99.0	99.2
Positive predictive value	4.3	4.9	3.5
Risk score cutoff: ≥19 (%)			
Sensitivity	36.7	36.1	30.4
Specificity	87.7	87.6	90.0
Negative predictive value	97.9	97.5	98.1
Positive predictive value	8.3	9.2	6.9

## DISCUSSION

When applying the baseline HE risk score to the UK cohort, the sensitivity and specificity in the primary analysis were comparable with those published by Tapper and colleagues in a predominately male US cohort. A sensitivity of ≥91% using a cutoff of ≥−11 was achieved. The specificity from applying the UK cohort to the HE risk scores was slightly lower with 86% in the UK compared to 91% in the US cohort, when using a cutoff of ≥27 points. The longitudinal HE risk scores were comparable between the UK and US cohorts with a sensitivity of 89% in the UK cohort, slightly less than the US cohort at 90% sensitivity when using a risk cutoff of ≥−3 and a specificity of 88% compared to the US cohort’s 91% when using a cutoff of ≥19 points.

The baseline characteristics in our study were reflective of those used in the US cohort analysis, with the exceptions of the UK cohort having a higher proportion of females (41%) and a higher proportion of patients receiving treatment with beta-blockers and statins. However, the proportion of patients developing HE in the UK cohort was significantly lower than in the US cohort (40% vs. 4% in the UK cohort). This may be due to the way that cases were selected or a differing clinical setting. Furthermore, HE is poorly recorded in the UK routine health care data since there is no specific ICD-10 code.

Several methods have been suggested in the prediction of developing HE, including psychometric testing, serum IL-6 levels, ammonia levels, quality of life scores, and genetics. While psychometric tools are the most widely used method for predicting HE, they are often challenging to implement and may not capture the entire risk profile of patients with cirrhosis developing HE. Similarly, Quality of life questionnaires require screening patients and relying on patients’ willingness to share information. Though levels of IL-6 and ammonia and alterations in the glutaminase gene have been reported as possible indicators of HE in patients with cirrhosis, they may not always be readily available within health care data sets.

There is currently no widely accepted screening method for HE in routine clinical practice; however, within this UK cohort, it is envisaged that the risk model could be used to assist in screening for patients likely to develop HE, where the sensitivity of the model becomes a key factor. The baseline HE risk score gives a 5-year risk of a patient developing HE at the point of liver cirrhosis diagnosis. Defining the medium-risk to high-risk groups as patients with a score of ≥−11 would predict 24% as being at low risk for HE (an NPV of 99% and a PPV of 5%). Having selected and excluded these lower-risk patients, those of a higher risk remain to be considered.

The longitudinal HE risk score provides a 1-year risk of HE for any given patient based on their most recent clinical parameters. The US cohort used a cutoff score of ≥−3 within the longitudinal HE risk score to achieve a minimum sensitivity of 90%. Using the HE risk score to stratify patients with cirrhosis from the UK for monitoring, considering those with risk scores ≥−3 as higher risk, 39% of the patients could be excluded from monitoring with a PPV of 4% and an NPV of 99%.

The baseline HE risk score cutoff value was based on a minimum sensitivity of 80%, which gave a cutoff at ≥−2, and achieved an overall sensitivity of 81% and a specificity of 37%. This would highlight patients at high risk, who require more intense monitoring, patient education of symptoms, and targeted questioning in clinical consultations. Within the longitudinal HE risk score, the minimum cutoff at >80% sensitivity was ≥2, which produced a sensitivity of 81%, a specificity of 54%, an NPV of 98.9%, and a PPV of 5%.

The AUC scores for baseline risk score were lower in the United Kingdom than the United States, the values were 0.58 and 0.68. For the longitudinal risk score, the AUC scores for the United Kingdom and United States were 0.64 and 0.73. Longitudinal risk scores were consistently higher for both the US and UK populations.

A key finding was that there was little difference between males and females when comparing the specificity and sensitivity in both risk scores (baseline sensitivity cutoff ≥−11: 92% male vs. 92% female; baseline specificity cutoff ≥27: 86% male vs. 87% female; longitudinal sensitivity cutoff ≥−3: 89% male vs. 86% female; longitudinal specificity cutoff ≥19: 88% male vs. 90% female). The HE risk scores were designed in a predominantly male population, so the similarity in performance provides evidence that the score can be successfully generalized to a wider population.

### Strengths/Limitations of the study

As CPRD contains data from routine practices, some data may be missing and coding inaccuracies may occur, leading to misclassification of disease. However, only patients who met CPRD research quality were included. One major limitation of testing the HE risk scores using UK data is the major underdiagnosis and/or coding of HE. HE is not currently well coded in the UK health care system, therefore the sensitivity and specificity values for false negatives should be interpreted with caution. However, the sensitivity of model analysis reported comparable results to the primary population with cirrhosis, which supports the decision to include patients with a prescription of rifaximin-α in the HE diagnosis population. While Tapper and colleagues included patients only with a cirrhosis code and an Aspartate aminotransferase-to-platelet ratio index score of >2, or a cirrhosis-related complication to improve specificity, this study only considered those with a prior cirrhosis code.

While the use of lactulose has been linked to the identification of patients with HE, it was not used as part of this study’s primary analysis. Investigations found that patients receiving lactulose after cirrhosis diagnosis differed from the population with HE in both mortality rates and prescribing patterns. Our sensitivity analysis using lactulose showed lower or the same AUC scores than the primary analysis. This could imply that either the model performance was lower than initially suggested in the primary analysis, using lactulose as a proxy for HE introduces false-positive HE diagnoses, or including a lactulose prescription had no effect on the model performance.

## CONCLUSIONS

When applying the risk scores to a UK cohort of patients with liver cirrhosis, the results were similar to those found by Tapper and colleagues in a male US cohort. Despite being developed in a predominantly US male cohort, the HE risk scores were reliable in selecting high-risk cases who were both male and female in a UK population.

## Supplementary Material

SUPPLEMENTARY MATERIAL
