# Supplementary material for: Predicting hepatic encephalopathy in patients with cirrhosis: A UK population–based study and validation of risk scores
Source: Hepatol Commun. 2023 Nov 6;7(11):e0307. doi: 10.1097/HC9.0000000000000307 (PMC10629733; doi:10.1097/HC9.0000000000000307)
Supplement: SUPPLEMENTARY MATERIAL [file hc9-7-e0307-s001.docx]

# Supplementary

## Figure S1 | Calculation of sensitivity, specificity, negative predictive value and positive predictive value.


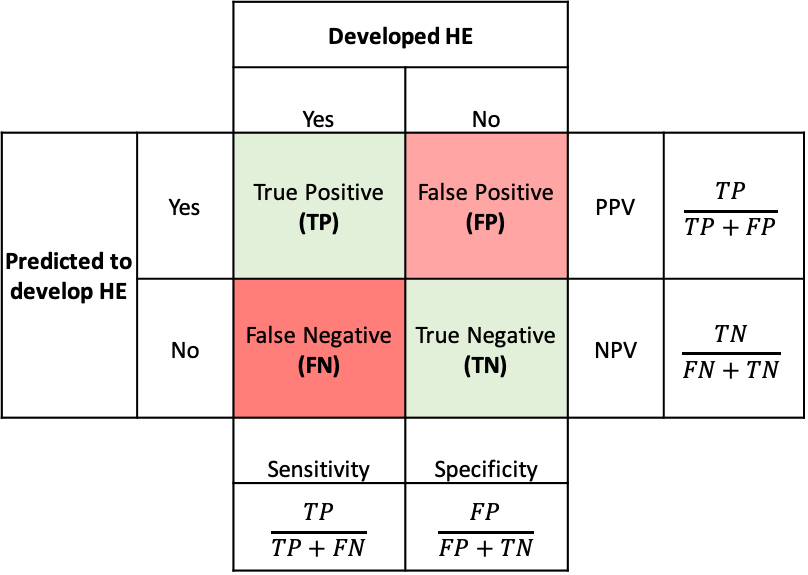


## Table S1 | Baseline characteristics for the sensitivity cohort

|  | Cirrhosis patients | | Developed HE | | No HE | | p-value |
| --- | --- | --- | --- | --- | --- | --- | --- |
| Total, n | 40,809 |  | 912 |  | 39,897 |  | <0.001 |
| Age (years) |  |  |  |  |  |  |  |
| Mean (SD) | 61.4 | (13.7) | 59.9 | (11.01) | 61.4 | (13.75) |  |
| Median (IQR) | 62.0 | (52.0-71.0) | 61.0 | (52.0-68.0) | 62.0 | (52.0-72.0) |  |
| Gender, n(%) | | | | | | | <0.001 |
| Male | 24,107 | (59.0%) | 602 | (66.0%) | 23,505 | (58.9%) |  |
| Female | 16,702 | (40.9%) | 310 | (34.0%) | 16,392 | (41.1%) |  |
| BMI, kg/m^2^ | | | | | | | <0.001 |
| Mean (SD) | 29.0 | (6.8) | 30.4 | (6.6) | 29.0 | (6.8) |  |
| Median (IQR) | 28.2 | (24.2-32.9) | 29.5 | (25.5-34.7) | 28.2 | (24.2-32.9) |  |
| Albumin g/dL | | | | | | | <0.001 |
| Mean (SD) | 3.6 | (0.7) | 3.25 | (0.6) | 3.6 | (0.7) |  |
| Median (IQR) | 3.7 | (3.1-4.1) | 3.30 | (2.8-3.7) | 3.7 | (3.1-4.1) |  |
| Bilirubin mg/dL | | | | | | | <0.001 |
| Mean (SD) | 2.0 | (3.3) | 2.9 | (3.7) | 2.0 | (3.2) |  |
| Median (IQR) | 0.9 | (0.6-1.9) | 1.8 | (1.1-3.2) | 0.9 | (0.5-1.9) |  |
| Beta blockers, n(%) | | | | | | | 0.009 |
| Yes | 11,277 | (27.6%) | 287 | (31.5%) | 10,990 | (27.6%) |  |
| No | 29,532 | (72.4%) | 625 | (68.5%) | 28,907 | (72.5%) |  |
| Statins, n(%) | | | | | | | 0.5 |
| Yes | 12,421 | (30.4%) | 286 | (31.4%) | 12,135 | (30.4%) |  |
| No | 28,388 | (69.6%) | 626 | (68.6%) | 27,762 | (69.6%) |  |
| Age categories, n(%) |  |  |  |  |  |  | <0.001 |
| Age 18-30 | 477 | (1.2%) | 2 | (0.2%) | 475 | (1.2%) |  |
| Age 31-40 | 2212 | (5.4%) | 35 | (3.8%) | 2177 | (5.5%) |  |
| Age 41-50 | 6432 | (15.8%) | 159 | (17.4%) | 6273 | (15.7%) |  |
| Age 51-60 | 10,062 | (24.7%) | 259 | (28.6%) | 9803 | (24.6%) |  |
| Age >60 | 21,626 | (53.0%) | 457 | (50.1%) | 21169 | (53.1%) |  |
| BMI categories, n(%) |  |  |  |  |  |  | 0.004 |
| Underweight | 413 | (1.0%) | 6 | (0.7%) | 407 | (1.0%) |  |
| Normal weight | 3905 | (9.6%) | 68 | (7.5%) | 3837 | (9.6%) |  |
| Overweight | 4558 | (11.2%) | 100 | (11.0%) | 4458 | (11.2%) |  |
| Obese | 5882 | (14.4%) | 167 | (18.3%) | 5715 | (14.3%) |  |
| Missing | 26,051 | (63.8%) | 571 | (62.6%) | 25,480 | (63.9%) |  |

HE = hepatic encephalopathy, QR = interquartile range, SD = standard deviation

|  | Baseline model | | Longitudinal model | |
| --- | --- | --- | --- | --- |
|  | Tapper model | UK model (Sensitivity cohort) | Tapper model | UK model (Sensitivity cohort) |
| Area under the curve | 0.68 | 0.59 | 0.73 | 0.65 |
| Sensitivity Cut off: | ≥-11 | ≥-11 | ≥-3 | ≥-3 |
| Sensitivity, (%) | 90.7% | 93.5% | 90.3% | 91.6% |
| Specificity, (%) | **--** | 23.9% | **--** | 39.6% |
| Negative predictive value, (%) |  | 99.4% |  | 99.6% |
| Positive predictive value, (%) |  | 2.7% |  | 2.7% |
| Specificity Cut off: | ≥27 | ≥27 | ≥19 | ≥19 |
| Sensitivity, (%) | **--** | 25.3% | **--** | 38.4% |
| Specificity, (%) | 91.2% | 86.0% | 90.6% | 87.4% |
| Negative predictive value, (%) |  | 98.1% |  | 98.7% |
| Positive predictive value, (%) |  | 4.0% |  | 5.3% |
| >80% Sensitivity Cut off: | -- | >-2 | -- | >2 |
| Sensitivity, (%) | -- | 84.2% | -- | 84.1% |
| Specificity, (%) | -- | 36.7% | -- | 53.6% |
| Negative predictive value, (%) |  | 99.0% |  | 99.5% |
| Positive predictive value, (%) |  | 3.0% |  | 3.2% |

## Table S2 | Risk model performance in the sensitivity cohort for baseline and longitudinal models compared to Tapper

## Table S3 | Baseline characteristics for the sensitivity cohort using lactulose

|  | Cirrhosis patients | | Developed HE | | No HE | | p-value |
| --- | --- | --- | --- | --- | --- | --- | --- |
| Total, n | 32,972 |  | 6,498 |  | 26,474 |  | <0.001 |
| Age (years) |  |  |  |  |  |  |  |
| Mean (SD) | 60.9 | (13.5) | 59.3 | (12.6) | 61.3 | (13.6) |  |
| Median (IQR) | 61 | (51.0-71.0) | 60 | (50.0-68.0) | 62.0 | (52.0-71.0) |  |
| Gender, n(%) | | | | | | | <0.001 |
| Male | 19,976 | (61%) | 4,106 | (63%) | 15,870 | (60%) |  |
| Female | 12,996 | (39%) | 2,392 | (37%) | 10,604 | (40%) |  |
| BMI, kg/m^2^ | | | | | | | 0.6 |
| Mean (SD) | 29.1 | (6.8) | 29 | (6.6) | 29.2 | (6.8) |  |
| Median (IQR) | 28.4 | (24.3-33.0) | 28.3 | (24.3-32.9) | 28.4 | (24.3-33.1) |  |
| Albumin g/dL | | | | | | | <0.001 |
| Mean (SD) | 3.6 | (0.7) | 3.4 | (0.7) | 3.7 | (0.7) |  |
| Median (IQR) | 3.7 | (3.1-4.1) | 3.4 | (2.9-3.9) | 3.7 | (3.2-4.2) |  |
| Bilirubin mg/dL |  |  |  |  |  |  | <0.001 |
| Mean (SD) | 2 | (3.2) | 2.7 | (3.8) | 1.8 | (3.1) |  |
| Median (IQR) | 0.9 | (0.6-1.9) | 1.4 | (0.8-3.0) | 0.9 | (0.5-1.7) |  |
| Beta blockers, n(%) | | | | | | | 0.026 |
| Yes | 8,896 | (27%) | 1,682 | (26%) | 7,214 | (27%) |  |
| No | 24,076 | (73%) | 4,816 | (74%) | 19,260 | (72%) |  |
| Statins, n(%) | | | | | | | <0.001 |
| Yes | 9,762 | (30%) | 1,697 | (26%) | 8,065 | (30%) |  |
| No | 23,210 | (70%) | 4,801 | (73%) | 18,409 | (69%) |  |
| Age categories, n(%) |  |  |  |  |  |  | <0.001 |
| Age 18-30 | 414 | (1.3%) | 52 | (0.8%) | 362 | (1.4%) |  |
| Age 31-40 | 1,837 | (5.6%) | 396 | (6.1%) | 1,441 | (5.4%) |  |
| Age 41-50 | 5,256 | (16%) | 1,225 | (19%) | 4,031 | (15%) |  |
| Age 51-60 | 8,402 | (25%) | 1,764 | (27%) | 6,638 | (25%) |  |
| Age >60 | 17,063 | (52%) | 3,061 | (47%) | 14,002 | (53%) |  |
| BMI categories, n(%) |  |  |  |  |  |  | <0.001 |
| Underweight | 298 | (0.9%) | 64 | (1.0%) | 234 | (0.9%) |  |
| Normal weight | 3,019 | (9.2%) | 533 | (8.2%) | 2,486 | (9.4%) |  |
| Overweight | 3,639 | (11%) | 687 | (11%) | 2,952 | (11%) |  |
| Obese | 4,760 | (14%) | 850 | (13%) | 3,910 | (15%) |  |
| Missing | 21,256 | (64%) | 4,364 | (67%) | 16,892 | (64%) |  |

HE = hepatic encephalopathy, QR = interquartile range, SD = standard deviation

## Table S4 | Risk model performance in the sensitivity cohort using lactulose for baseline and longitudinal models compared to Tapper

|  | Baseline model | | Longitudinal model | |
| --- | --- | --- | --- | --- |
|  | Tapper model | UK model (Sensitivity cohort) | Tapper model | UK model (Sensitivity cohort) |
| Area under the curve | 0.68 | 0.58 | 0.73 | 0.59 |
| Sensitivity Cut off: | ≥-11 | ≥-11 | ≥-3 | ≥-3 |
| Sensitivity, (%) | 90.7% | 86.1% | 90.3% | 75.3% |
| Specificity, (%) | **--** | 26.3% | **--** | 41.4% |
| Negative predictive value, (%) |  | 88.6% |  | 91.8% |
| Positive predictive value, (%) |  | 22.3% |  | 16.2% |
| Specificity Cut off: | ≥27 | ≥27 | ≥19 | ≥19 |
| Sensitivity, (%) | **--** | 23.1% | **--** | 24.7% |
| Specificity, (%) | 91.2% | 87.8% | 90.6% | 88.7% |
| Negative predictive value, (%) |  | 82.3% |  | 88.7% |
| Positive predictive value, (%) |  | 31.7% |  | 24.7% |
| >80% Sensitivity Cut off: | -- | >-6 | -- | >-5 |
| Sensitivity, (%) | -- | 80.0% | -- | 81.1% |
| Specificity, (%) | -- | 33.8% | -- | 33.8% |
| Negative predictive value, (%) |  | 87.3% |  | 92.2% |
| Positive predictive value, (%) |  | 22.9% |  | 15.5% |
